# Supplementary material for: SPOT-RASTR—A cryo-EM specimen preparation technique that overcomes problems with preferred orientation and the air/water interface
Source: PNAS Nexus. 2024 Aug 6;3(8):pgae284. doi: 10.1093/pnasnexus/pgae284 (PMC11303004; doi:10.1093/pnasnexus/pgae284)
Supplement: pgae284_Supplementary_Data [file pgae284_supplementary_data.zip › Supplementals.pdf]

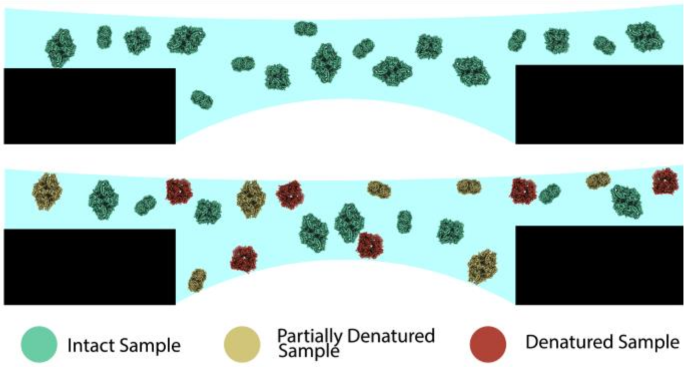

Figure S1. Top) Expected well dispersed β-gal particles in vitreous ice. Bottom) β-gal dispersion in vitreous ice considering the air-water interface absorption of the particles.

Video1. Selected tomogram showing two tubes with decorated β-gal.

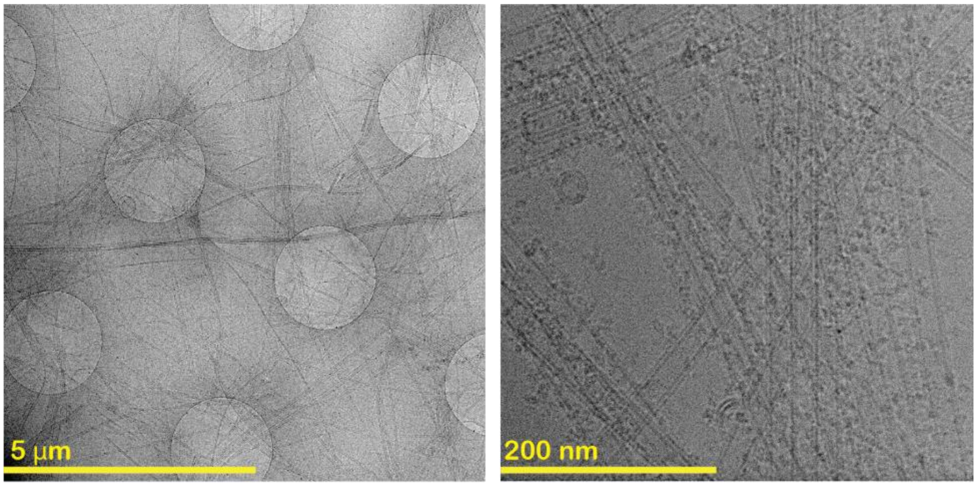

Figure S2. Left) Hole view of the decorated tubes with β-gal showing the huge aggregates due to the premixing. Right) The exposure from one of the previous holes showing the tube aggregate caused by the β-gal multi his-tag

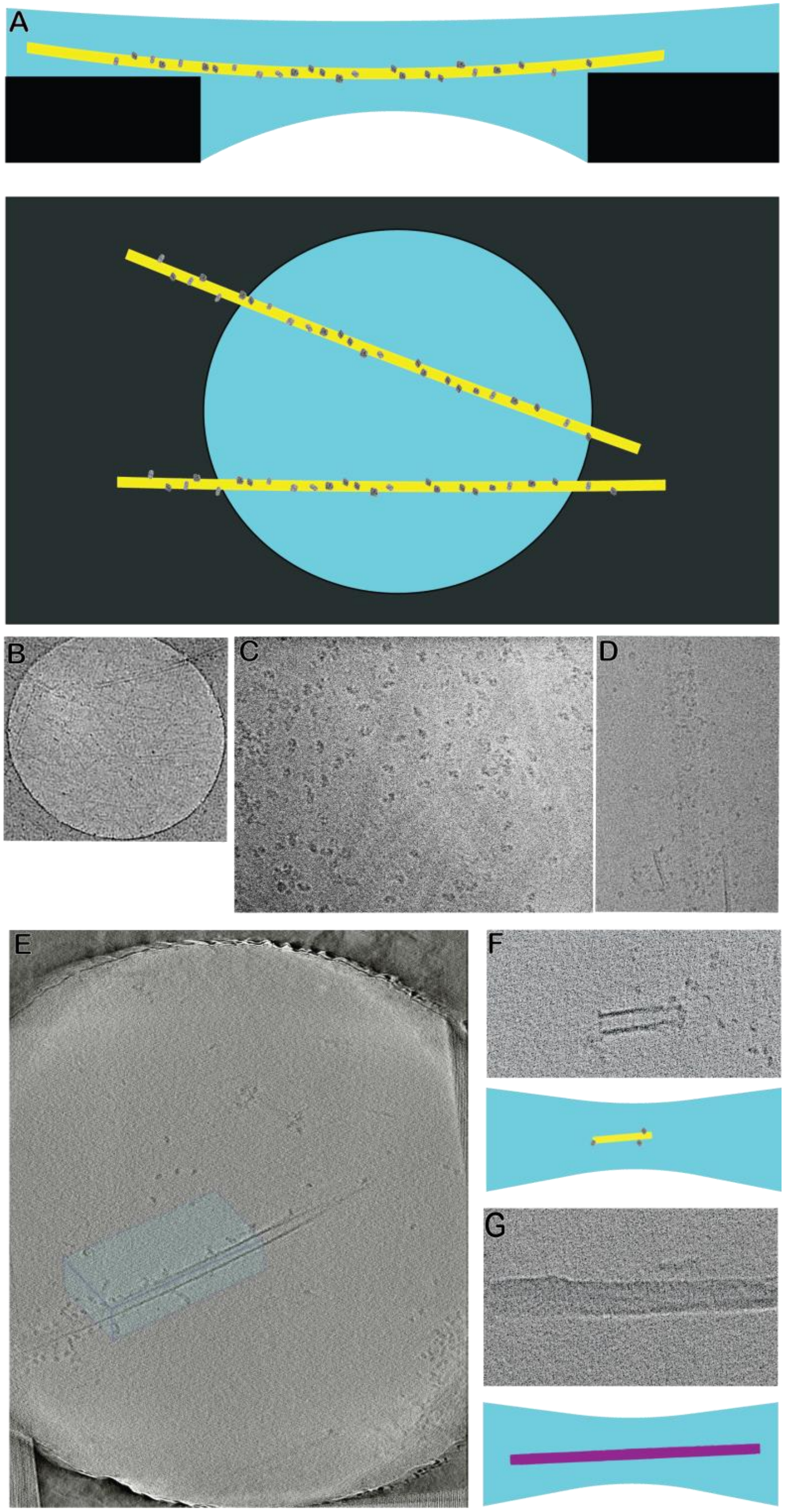

Figure S3. A) Schematic formation of the decorated tube on the cryo-EM grid hole. B) A crowded ghost tubes region hole view. C) Crowded ghost tubes exposure example decorated with β-gal. D) Crowded ghost tubes exposure example decorated with SiRFP/HP. E) A tomogram slice of the decorated tube which its schematic has shown in the Fig.2. F) Short tube decorated with β-gal. G) A dark tube with no decoration.

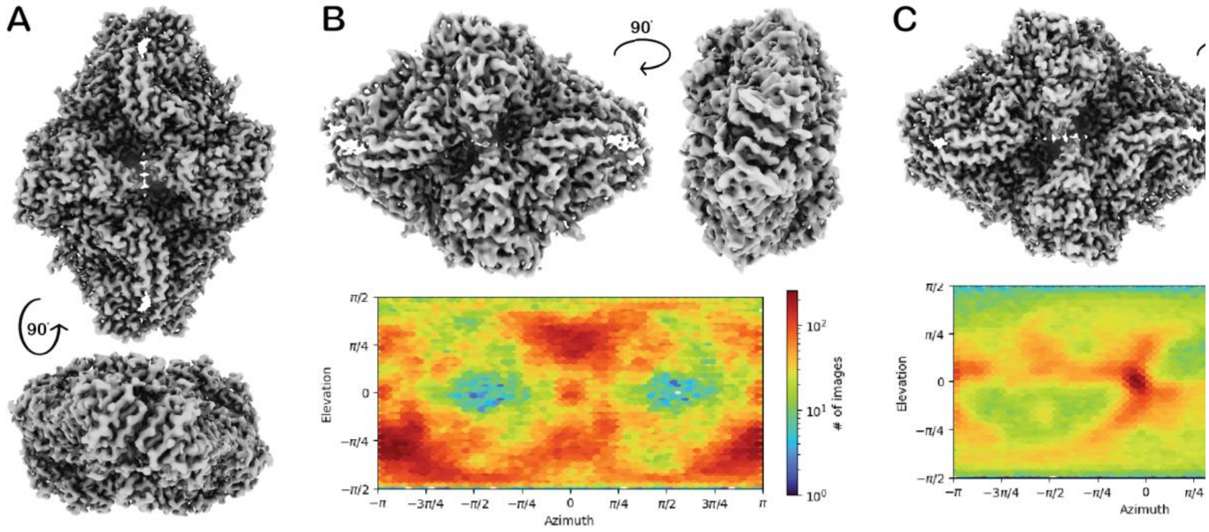

Figure S4. A) 3D reconstruction from β-gal sample data which was prepared with conventional blotting with D2 symmetry applied. B) 3D map and particles orientation distribution

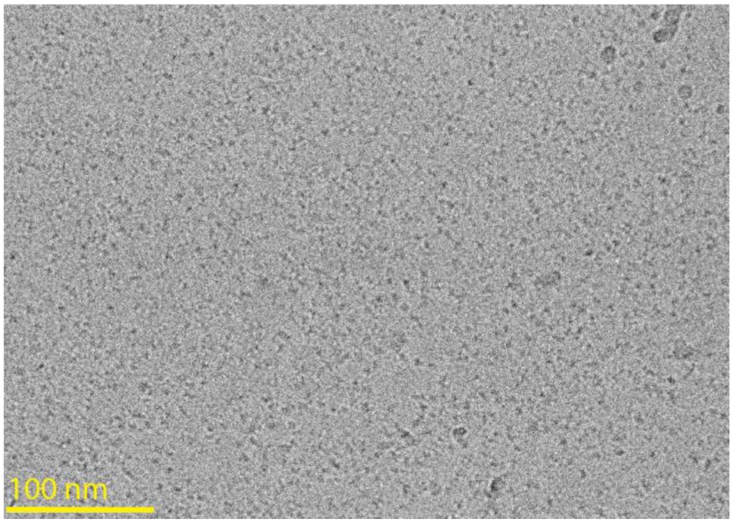

Figure S5. Disassembled SiRFP/HP monomers falling apart, not showing any dimer formation.
