# Supplementary figures and images for: SPOT-RASTR—A cryo-EM specimen preparation technique that overcomes problems with preferred orientation and the air/water interface
Source: PNAS Nexus. 2024 Aug 6;3(8):pgae284. doi: 10.1093/pnasnexus/pgae284 (PMC11303004; doi:10.1093/pnasnexus/pgae284)

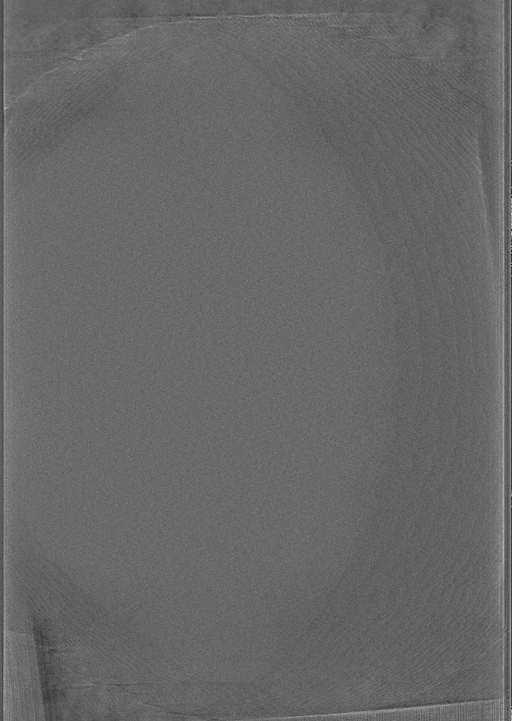

Supplement: pgae284_Supplementary_Data [file pgae284_supplementary_data.zip › PNASNEXUS-PNASNEXUS-2024-00184-TR-s01.gif]
